# People quasi-randomly assigned to farm rice are more collectivistic than people assigned to farm wheat

**DOI:** 10.1038/s41467-024-44770-w

**Published:** 2024-02-27

**Authors:** Thomas Talhelm, Xiawei Dong

**Affiliations:** 1 University of Chicago Booth School of Business, Chicago, IL USA; 2 Hong Kong University of Science and Technology, Hong Kong, China

**Keywords:** Human behaviour, Culture, Decision making

## Abstract

The rice theory of culture argues that the high labor demands and interdependent irrigation networks of paddy rice farming makes cultures more collectivistic than wheat-farming cultures. Despite prior evidence, proving causality is difficult because people are not randomly assigned to farm rice. In this study, we take advantage of a unique time when the Chinese government quasi-randomly assigned people to farm rice or wheat in two state farms that are otherwise nearly identical. The rice farmers show less individualism, more loyalty/nepotism toward a friend over a stranger, and more relational thought style. These results rule out confounds in tests of the rice theory, such as temperature, latitude, and historical events. The differences suggest rice-wheat cultural differences can form in a single generation.

## Abstract (web summary)

According to the rice theory, the demands of rice farming might make cultures more collectivistic. Here the authors provide evidence in support of this theory by showing that Chinese farmers who were quasi-randomly assigned to farm rice score higher on measures related to collectivism than those assigned to farm wheat.

## Introduction

The rice theory of culture argues that traditional paddy rice farming caused rice-farming cultures to become more collectivistic than wheat-farming cultures^1,2^. Paddy rice required irrigation networks and labor demands double those of wheat, which tied farmers together in tight, interdependent relationships. Rice cultures from Japan to Sierra Leone formed cooperative labor exchanges to cope with the crushing labor demands of rice^2,3^. Despite prior evidence^1,4,5^, causality is a difficult question for this theory. Does rice farming actually cause these cultural differences?

When we compare cultures, third variables make it difficult to know what is a cause and what is just a correlation. Experiments solve this problem, but in the case of rice and wheat, we cannot randomly assign people to farm rice or wheat for years on end. Nor is it easy to simulate the complicated process of rice farming in a laboratory. It is essentially impossible to bring participants to a lab and simulate the full scope of social relationships, monitoring, and reciprocity that stretch over seasons, years, and generations in rice villages.

**Fig. 1:**
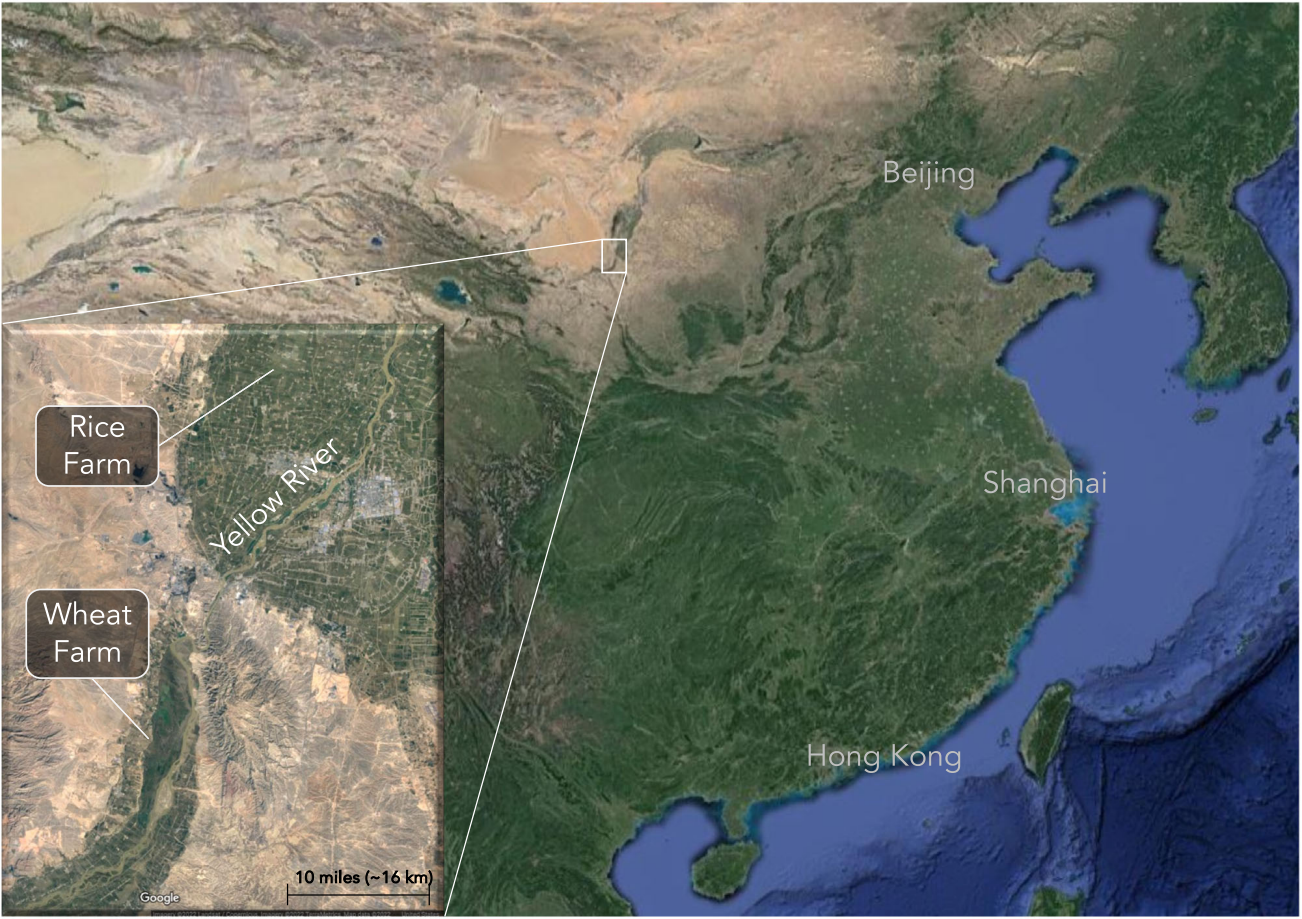
The location of the two state-owned farms in Ningxia, China. Although the two state farms are only 56 kilometers from each other, a small difference in topography allows the rice farm (Lianhu) to farm paddy rice. In contrast, much of the land in the wheat farm (Qukou) is 50–100 m above the nearby Yellow River, making large-scale irrigation uneconomical. The soil on the wheat farm is also sandier, which makes it difficult to retain water in rice fields. Image from Google Earth.

This study takes advantage of a unique time in history when the Chinese Communist Party essentially randomly assigned people to farm rice or wheat. After World War II, the government created state farms around the country to open up new farmland, put former soldiers to work, and re-educate urban youth^6^. In northern Ningxia Province, the government created two farms just 56 kilometers from each other—one rice, one wheat (Fig. 1).

The Lianhu rice farm and Qukou (“choo-koh”) wheat farm have nearly identical environments. They have similar temperature, rainfall, and acreage (Table 1). Both sit near the Yellow River, which brings water that can irrigate paddy rice. But a minor difference in the topography allowed one to farm rice and the other wheat. Most of the wheat farm is 50 to 100 meters above the river, which prevents economical irrigation. This created a rare natural experiment where Chinese citizens were quasi-randomly assigned to farm rice or wheat.

**Table 1:** The rice and wheat state farms have nearly identical natural environments

	Lianhu rice farm	Qukou wheat farm
Average July temperature	23.6 °C	23.0 °C
Average January temperature	−7.6 °C	−7.7 °C
Average precipitation	181 mm	171 mm
Farm size	990 Hectares	1180 Hectares
Elevation	1120–1125 m	1156–1700 m
Animal economy percentage (%)	12	8
Year founded	1955	1956

These two state farms in Ningxia Province are 56 kilometers away from each other and functionally equivalent in factors like temperature, precipitation, and size. However, the wheat farm has sandier soil and slightly higher elevation than the nearby Yellow River, which makes irrigation impractical.

In this study, we test for cultural differences by comparing the rice and the wheat farm. These two farms provide a situation close to a naturalistic randomized experiment, which helps rule out potential confounds. If the rice theory is true, we should see cultural differences between the rice and wheat farms that mirror larger comparisons between China’s rice-farming south and wheat-farming north.

**Fig. 2:**
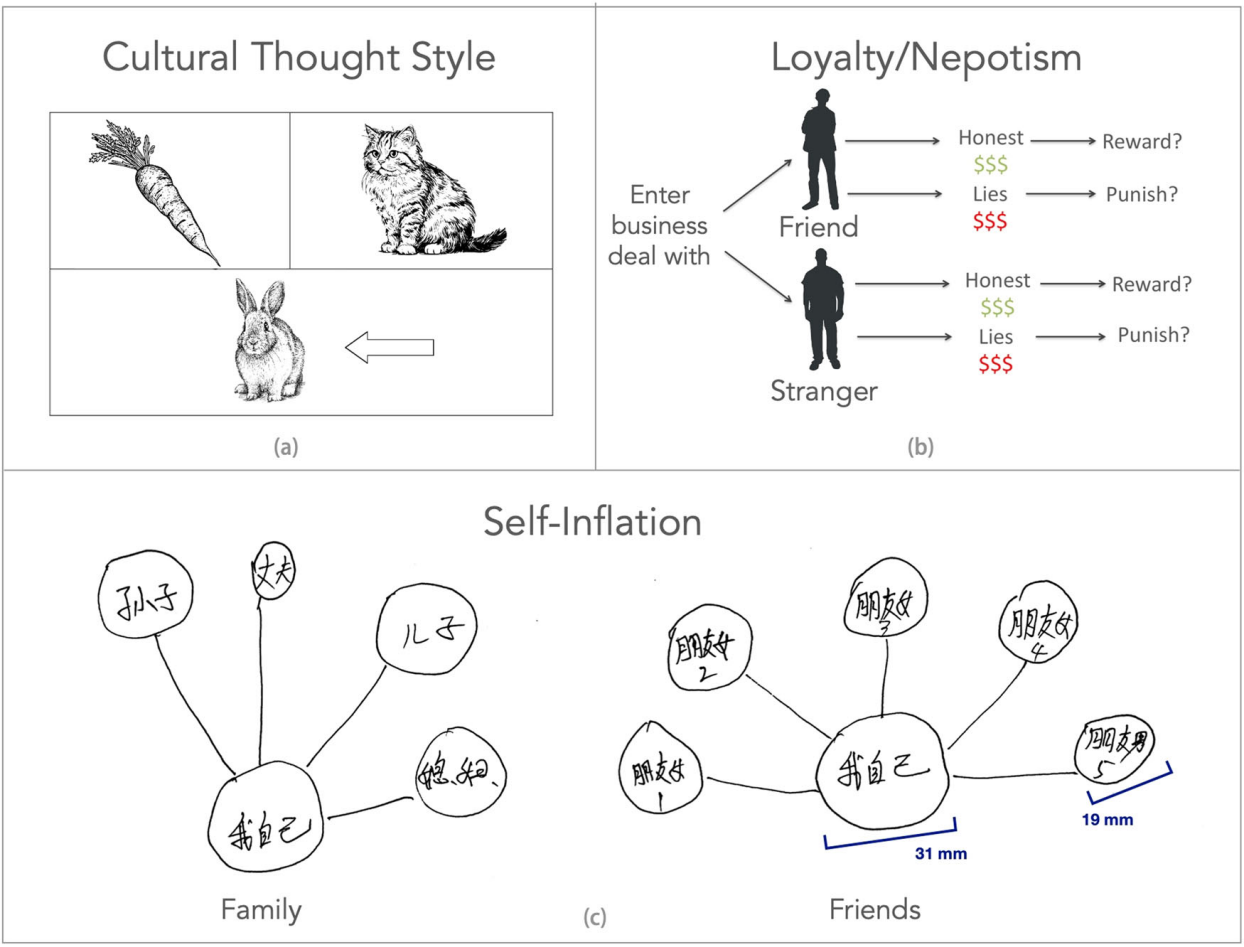
The Psychological Measures of Thought Style, Loyalty/Nepotism, and Implicit Individualism. **a** The triad categorization task (top left) measures holistic thought, which is more common in collectivistic cultures^1,23^. Participants choose one of two items (carrot or cat) to pair with an object (rabbit). Rabbit and carrot are a relational (holistic) pairing, whereas rabbit and cat are a categorical (analytic) pairing. **b** In the loyalty/nepotism task (top right), participants read scenarios about doing a business deal with a friend or a stranger^1,18^. Then they find out the friend/stranger was honest or dishonest. They can reward the friend or stranger for their honesty, and they can punish them for their dishonesty. We measured loyalty/nepotism as whether they treated the friend better than the stranger, even though their behavior was the same. **c** The sociogram task (bottom) measures implicit individualism. Participants draw circles to represent themselves and their family (left) or friends (right). We measured whether people draw the self larger than they draw others. Previous studies found that people in individualistic cultures like the US self-inflate more than people in collectivistic cultures like Japan^1,7,17^. Thought style drawings are from doublebubble_rus (rabbit) and Artem (cat and carrot) at Adobe Stock – stock.adobe.com. The human figure outlines are from rejon at Wikimedia Commons – commons.wikimedia.org. All uses must include the credit line shown on the site and contained in the IPTC credit line field of the file, for example “Agency Name/Contributor Name – stock.adobe.com”.

We tested farmers on three measures that have shown rice-wheat differences in China in previous studies^1,7,8^. In a previous study, we tested farmers on these two farms for social comparison, finding that rice farmers report more social comparison than wheat farmers^9^. In this new study, we tested people on psychological traits associated with collectivism. We used measures of implicit individualism on a sociogram-drawing task, loyalty/nepotism toward a close friend, and a measure of relational/holistic cultural thought style, which tends to be more common in collectivistic cultures^1,10^. Figure 2 explains the tasks.

The results showed more cultural markers of collectivism among farmers on the rice farm than the wheat farm. Rice farmers showed less implicit individualism on the sociogram task, more loyalty/nepotism, and more holistic thought than wheat farmers. These differences were not explained by any of the demographic characteristics we measured.

## Results

### No evidence of self-selection

If these two farms are truly an unintentional quasi-randomized experiment, they would offer a much stronger test of the rice theory than previous research^1,4^. But one threat to the natural experiment is if people *chose* to move to one farm over the other. For example, if people knew (or even just had a vague sense) that rice requires working more closely with other people, then the people who already liked working with other people might actively choose to go to the rice farm rather than the wheat farm. Another example of selection would be if the government intentionally selected people with prior experience farming rice for the rice farm. These scenarios would invalidate the natural experiment.

Yet historical records^11,12^ and our interviews with farm administrators suggest that government assignments between these two rice and wheat farms were effectively random. The government was not concerned with modern ideas of occupational interest questionnaires or personal choice. Farm records reveal that most farmers in Lianhu and Qukou were (a) regular citizens assigned by the government, (b) military veterans assigned by the government to the area, or (c) educated youth assigned to Ningxia during the Cultural Revolution^11,12^.

There is a telling example from 1960, when the government started the Youth Assisting Ningxia (支宁青年) movement. This movement sent youngsters to Ningxia to develop new farmland. The rice-farming province of Zhejiang sent many youth to Ningxia for this movement. If leaders were trying to assign people to the right farm based on their skills, this was a golden opportunity to send people from rice areas to the rice farm. Yet records show they assigned nearly identical numbers of Youth Assisting Ningxia to the rice farm and the wheat farm^11,12^.

Later, the top-down assignments of the Cultural Revolution sent many youth to the countryside with very little preparation or forethought to placing the right individual in the right area^13^. The heritages of the farmers we tested are consistent with the idea that there was no selection based on people’s regional background. Among the 234 farmers we tested, only one was born in a majority rice province. The vast majority of farmers (> 99%) and their parents (98%) were from majority-wheat provinces on both farms.

### Nearly identical environments

Another type of threat is non-random selection of the locations. This could happen if the government chose to put the farms on top of villages that were already farming rice or wheat. If so, the farms could simply be carrying on the cultures of the areas.

However, historical records show this was not the case. One of the goals of the government’s push to create state farms was to open up new land to farming. Before the rice farm was created, the area was an “uninhabited” wetland with 72 lakes^11^.

The land on the wheat farm was also mostly un-farmed before the government founded the state farm. Much of the area was poorly suited to farming because it had desert soil (sierozem), which is dry and devoid of nutrients (< 1% organic matter). Farming only became widespread after government investment brought infrastructure and fertilizer into the area^12^. The lack of farming history on this land is important for the natural experiment because the two areas were relative blank slates for farming, rather than simply carrying on the farming practices of the area.

### No evidence of policy differences

Finally, another potential threat to the experiment is if the two farms had systematic differences in top-down policies or economic systems. China’s biggest policy change of the last 50 years started in 1978, when China started moving from the system of collective farming profits to letting individual families keep their own profits (包产到户). If one farm rolled out this system earlier than the other, this could have differential effects on the farms separate from rice and wheat.

However, the two farms belonged to the same land reclamation group (农垦集团). This shared bureaucracy meant the two farms had the same profit structures and incentives. The shared bureaucracy also meant they tended to roll out policies at the same time. For example, historical records show the two farms rolled out the shift to individual profits within months of each other^11,12^.

This quasi-random assignment offers the chance to test for rice-wheat cultural differences in a place where systematic genetic differences are unlikely. This is important because researchers have argued that genetic differences can at least partially explain East-West differences in collectivism^14^. Within China, there are genetic differences between people in rice-farming versus wheat-farming areas^15^, and there are physical differences between northerners and southerners^16^.

### Implicit Individualism: self-Inflation

The farmers were similar on most demographics (Table 2), and we used propensity score matching to equate the two samples on any remaining differences.

**Table 2:** Farmer sample demographics

Farm	*N*	Age (SD)	Male (%)	Family income ≤ US$444/month (%)^a^	Education ≤ high school (%)	Mother no formal education (%)
Wheat (Qukou)	130	47.6 (5.6)	66.7	92.3	92.2	64.8
Rice (Lianhu)	104	46.2 (5.7)	54.8	95.2	95.2	72.4

^a^Income is converted from 3,000 RMB to USD at the average exchange rate in 2017. Farmers reported their family monthly income per person in categories from 1 (1000 Yuan or less) to 11 (Over 10,000 Yuan). Participants reported their gender identity after the prompt “Gender” with options “male” and “female.”

Table 3 reports regression results.

**Table 3:** People on the rice farm self-inflated less, were more loyal/nepotistic to a friend, and thought more holistically than people on a nearby wheat farm

	*B*	SE	*t*	*P*	95% CI	
Self-inflation (family)						
Female	−0.46	0.72	−0.63	0.527	−1.88	0.97
Age	−0.08	0.07	−1.15	0.252	−0.20	0.05
Hui	−1.62	1.38	−1.17	0.242	−4.35	1.10
Family income	−0.24	0.37	−0.65	0.514	−0.97	0.49
Mother education	−0.54	0.60	−0.90	0.367	−1.72	0.64
Rice farm	−1.92	0.73	−2.62	0.010	−3.36	−0.47
Self-inflation (friends)						
Female	−0.14	0.72	−0.19	0.852	−1.55	1.28
Age	0.03	0.07	0.50	0.618	−0.10	0.16
Hui	1.09	1.42	0.77	0.445	−1.69	3.86
Family income	0.00	0.39	0.01	0.992	−0.76	0.77
Mother education	0.18	0.61	0.29	0.774	−1.02	1.37
Rice farm	−0.65	0.73	−0.89	0.377	−2.09	0.79
Loyalty/nepotism						
Female	1.21	0.68	1.77	0.078	−0.14	2.56
Age	−0.04	0.06	−0.71	0.477	−0.17	0.08
Hui	0.16	1.31	0.12	0.903	−2.43	2.75
Family income	−0.57	0.35	−1.61	0.108	−1.26	0.13
Mother education	−1.25	0.57	−2.20	0.029	−2.38	−0.13
Rice farm	1.83	0.70	2.63	0.009	0.46	3.20
Holistic thought						
Female	0.03	0.12	0.23	0.821	−0.21	0.27
Age	0.07	0.01	6.32	<0.001	0.05	0.09
Hui	−0.30	0.23	−1.29	0.199	−0.74	0.17
Family income	0.05	0.07	0.78	0.437	−0.08	0.19
Mother education	0.28	0.11	2.60	0.009	0.07	0.50
Rice farm	0.28	0.13	2.24	0.025	0.04	0.53

Rice and wheat farm samples are matched using propensity score matching. Female, Hui, and Rice Farm are dummy variables where 0 = no, 1 = yes. Age is in years. Hui are a Muslim religious group in China sometimes considered an ethnicity. Farmers reported monthly family income per person from 1 (*1000 Yuan and below*) to 11 (*10,000 Yuan and above*). Degrees of freedom = 189, except for self-inflation (friends) = 183 and holistic thought = 181 due to missing data. Analyses are regressions with two-tailed *p* values.

**Fig. 3:**
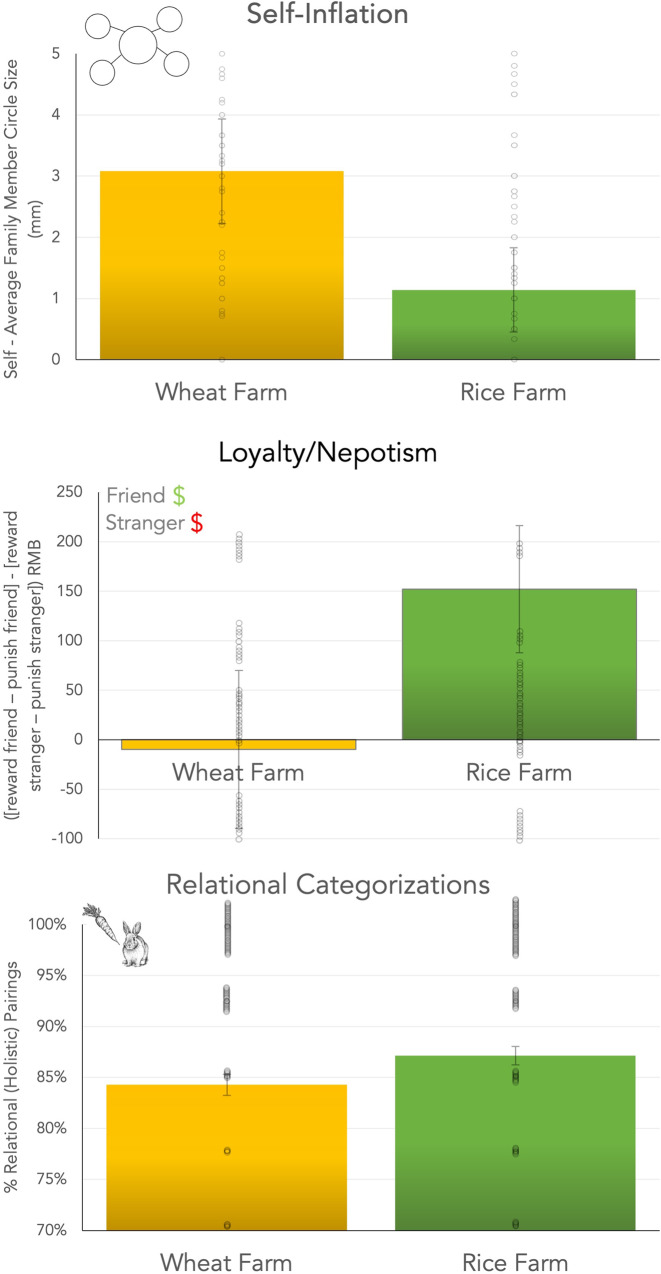
Rice Farmers Self-Inflated Less, Were More Loyal/Nepotistic, and Thought More Holistically than Nearby Wheat Farmers. Farmers quasi-randomly assigned to farm rice showed three hallmarks of collectivistic cultures compared to a nearby wheat farm. Rice farmers self-inflated less while drawing sociograms (top), were more loyal/nepotistic to friends (middle), and thought more holistically (bottom). Samples were *N* = 196, except the thought task (*N* = 188) due to missing data. Bars = 1 SEM. Dots = individual participants, displaced to avoid overlapping. Thought style drawings are from doublebubble_rus (rabbit) and Artem (carrot) at Adobe Stock – stock.adobe.com. All uses must include the credit line shown on the site and contained in the IPTC credit line field of the file, for example “Agency Name/Contributor Name – stock.adobe.com”.

Participants in the rice farm self-inflated less than participants in the wheat farm when they drew family members, (*B* = −1.92, *t*[189] = −2.62, *P* = 0.010, 95% CI [−3.36, −0.47], *r* = 0.18; Fig. 3). The pattern was similar when the farmers drew friends, but the difference was not significant, *B* = −0.65, *t*(183) = −0.89, *P* = 0.377, 95% CI [−2.09, 0.79], *P* = 0.242, *r* = 0.07. This could reflect the fact that some farmers had a hard time thinking of friends, suggesting that friend are not a relevant social category for farmers in China.

The self-inflation differences between the two farms were similar to a study of students from rice and wheat areas of China^7^. For comparison, this difference was about two-thirds the size of the difference in a previous study comparing people in the UK and Japan^17^. Thus, the difference between these two farms was smaller than East-West differences.

### Loyalty/nepotism

Rice farmers were more loyal/nepotistic than wheat farmers, *B* = 1.83, *t*(189) = 2.63, *P* = 0.009, 95% CI [0.46, 3.20], *r* = 0.18. Among the four scenarios, the biggest difference between the rice and wheat farms was in how they treated the stranger. Rice farmers punished the stranger more harshly than the wheat farmers, *B* = 1.27, *t*(189) = 2.66, *P* = 0.009, 95% CI [0.33, 2.22], *r* = 0.19.

We compared these differences to a previous study testing students in Singapore and the US^18^. In that study, the difference between the US and Singapore was $50. The difference between the rice and wheat farms was $24 (converted into USD). Thus, the difference between rice and wheat farms was roughly half the East-West difference.

### Holistic thought

The rice farmers thought more holistically on the categorization task than the wheat farmers, *B* = 0.28, *t*(181) = 2.24, *P* = 0.025, 95% CI [0.04,0.53], *r* = 0.15. These farmers were by far the most holistic sample we have ever tested. For example, in our prior studies, Chinese college students often choose 75% relational pairings, and US students choose 60% relational pairings^1^. The farmers chose nearly 90% relational pairings, which is the highest we have seen among the thousands of people who have taken this test in our studies and the studies of other researchers^1,10,19^. The farmers in China thought even more holistically than farmers in Turkey (68% relational pairings, Supplementary Note 4^19^). However, we can only make descriptive comparisons with Turkey. We could not make a statistical comparison between the farmers in China and Turkey because the earlier study did not report standard errors.

### Controlling for demographic differences

We used regressions to control for demographic differences between the rice and wheat farm samples in gender, age, income, religion, and maternal educational attainment. There were some significant demographic differences on the cultural tasks. Older farmers thought more holistically than younger farmers, *B* = 0.07, *t*(181) = 6.32, *P* < 0.001, 95% CI [0.05, 0.09], *r* = 0.44. Farmers whose mothers had higher educational attainment were less loyal/nepotistic, *B* = −1.25, *t*(189) = −2.20, *P* = 0.029, 95% CI [−2.38, −0.13], *r* = −0.24. However, rice-wheat differences held despite controlling for these demographic characteristics. This result makes it less likely that differences between the rice and wheat farms were due to sampling differences.

### Differences persist even among farmers not farming rice this year

The rice farm presents a unique opportunity to test whether the rice-wheat differences require direct, recent experience farming rice. In 2000, the rice farm started facing shortages of irrigation water, so it started a rotation program so that about one-third of farmers would farm dryland crops every year. About one-third (38.4%) of the participants on the rice farm did not farm rice in the year we collected data.

Importantly, the farm rotates farmers evenly year after year. That means this is a top-down assignment that applies evenly to all farmers. The farmers are not self-selected into the program.

This allows us to compare farmers who had direct experience farming rice in the year we collected data versus those who were farming dryland crops like wheat for the year. If direct experience is required to maintain these cultural differences, we should find that individual farmers’ rice experience in the year of the testing predicts cultural differences. For the farmers temporarily rotated away from rice, the experience farming wheat and other dryland crops should make them more individualistic.

The other possibility is that direct, recent experience is not required for rice-wheat differences. Instead, rice-wheat differences may be the result of accumulated experience—and shared through broader community norms and practices. If that is the case, then direct experience in any particular year should have little or no effect on rice-wheat cultural differences. We tested this by adding individual farmers’ percentage of land farmed with rice in the most recent year of farming (Table 4). The results clearly refuted the idea that recent individual experience farming rice is necessary for rice-wheat differences. Individual rice-farming percentages did not predict any of the cultural differences. Meanwhile, farm-level rice-wheat differences from Table 3 remained robust even after adding individual rice farming in Table 4. In sum, rice-wheat differences persisted, even among farmers who did not farm rice in the year we tested them.

**Table 4:** Community rice farming predicts cultural differences better than individuals’ direct experience in the year of the study

	*B*	SE	*t*	*P*	95% CI	
Self-inflation (family)						
Female	−0.11	0.72	−0.15	0.884	−1.52	1.31
Age	−0.04	0.07	−0.57	0.568	−0.17	0.09
Hui	−1.56	1.39	−1.12	0.263	−4.29	1.17
Family income	−0.10	0.39	−0.26	0.796	−0.86	0.66
Mother education	−0.63	0.59	−1.06	0.289	−1.80	0.53
Individual rice %	−0.31	0.97	−0.32	0.752	−2.22	1.60
Rice farm	−2.02	0.74	−2.71	0.007	−3.48	−0.56
Self-inflation (friends)						
Female	−0.25	0.71	−0.36	0.720	−1.64	1.13
Age	0.04	0.06	0.59	0.559	−0.09	0.16
Hui	1.09	1.39	0.78	0.437	−1.65	3.82
Family income	0.14	0.38	0.38	0.704	−0.60	0.89
Mother education	−0.08	0.58	−0.13	0.895	−1.22	1.07
Individual rice %	−0.67	0.94	−0.72	0.476	−2.53	1.18
Rice farm	−0.76	0.72	−1.06	0.292	−2.18	0.65
Loyalty/nepotism						
Female	1.21	0.69	1.77	0.078	−0.14	2.56
Age	−0.04	0.06	−0.70	0.483	−0.17	0.08
Hui	0.18	1.32	0.13	0.893	−2.43	2.79
Family income	−0.57	0.35	−1.61	0.109	−1.27	0.13
Mother education	−1.25	0.57	−2.18	0.031	−2.38	−0.12
Individual rice %	−0.12	0.93	−0.13	0.894	−1.95	1.71
Rice farm	1.85	0.71	2.61	0.010	0.45	3.24
Holistic thought						
Female	0.06	0.12	0.52	0.605	−0.18	0.30
Age	0.07	0.01	6.20	<0.001	0.05	0.09
Hui	−0.29	0.23	−1.23	0.219	−0.73	0.19
Family income	−0.01	0.06	−0.17	0.865	−0.13	0.12
Mother education	0.34	0.11	3.10	0.002	0.13	0.55
Individual rice %	0.07	0.17	0.42	0.675	−0.25	0.40
Rice farm	0.33	0.13	2.61	0.009	0.08	0.58

The state rice farm rotates a third of farmers every year to plant dryland crops to preserve limited irrigation water. This table tests whether farmers’ recent experience farming rice is more important (Individual Rice %) than the overall rice or wheat system of the farms (Rice Farm). Individual Rice % is the percentage of land that each farmer planted with rice in the most recent farming season. Farm samples are matched using propensity score matching. Female, Hui, and Rice Farm are dummy variables where 0 = no, 1 = yes. Degrees of freedom = 188, except for self-inflation (friends) = 182 and holistic thought = 180. Analyses are regressions with two-tailed *p* values.

## Discussion

Farmers on the state rice farm showed more hallmarks of collectivistic culture than farmers on the nearby state wheat farm. Rice farmers were more loyal/nepotistic toward friends, thought more holistically, and showed less implicit individualism than the nearby wheat farmers.

Because the Chinese government unintentionally created a quasi-random assignment experiment, these two farms provide a much cleaner test of causality than prior research. The fact that these two farms still showed cultural differences effectively rules out major potential confounds like temperature, latitude, and the many historical events that differed between northern and southern China.

The natural experiment value of these two farms can also provide insights into cultural differences besides collectivism. For example, one year later, we returned to the farms for a separate study on social comparison and happiness^9^. In that study, we tested whether social comparison is more common on the rice farm than the wheat farmers. The rice farmers reported more social comparison, which mirrored differences between China’s larger rice and wheat areas. That increased social comparison could explain why collectivistic cultures (and China’s rice regions^9^) tend to be less happy than individualistic cultures, even at the same level of wealth.

Rice-wheat differences persisted even among a subset of farmers on the rice farm who did not farm rice in the year we tested them. This suggests that there is an accumulated cultural pattern that does not require direct, recent experience. This fits with the fact that studies have found rice-wheat differences among people who have never worked as farmers, such as university students^1^ and Starbucks customers in China’s biggest cities^20^. Rice culture does not disappear as soon as people put down their plows.

### Limitations

One limitation of this study is that there could be demographic differences, such as age and educational attainment between the two farms. Another related limitation is that we could not randomly sample farmers because of the difficulty of reaching participants in these remote farms. To make up for this, we took three steps:

We (1) measured a range of demographic variables for the farmers to measure variables that might otherwise be hidden confounds, (2) statistically controlled for demographics in generalized linear models, and (3) used propensity score matching to create sub-samples from the rice and wheat farms that minimized potential demographic confounds. These steps help lessen the possibility that demographic differences are causing spurious differences between the farms.

A second limitation is that the farmers had some difficulty completing the tasks that were originally designed for college students. Some farmers had difficulty bringing to mind friends on the sociogram task, which led us to create a family version of the task. We are making the family sociogram task available to other researchers to use (available on the OSF page).

Third, the low reliability of farmers’ responses to the relational mobility questionnaire suggests that they had trouble answering these questions. The relational mobility questionnaire uses complex and negatively worded questions, which may be hard for people to process. Thus, we were forced to discard this questionnaire. These difficulties speak to the importance of creating simple, concrete tasks like the triad categorization task or the new family sociogram task that are useful across diverse populations.

### From two farms to a larger theory of culture

This study presents a tightly controlled, narrow comparison of rice and wheat. Yet the findings imply that there is a more general theory of the causes of cultural differences. The larger idea is that interdependence in subsistence style (or perhaps in work more generally) makes cultures more collectivistic. For example, cultures that used irrigation tend to be more collectivistic today^5^.

Rice and wheat farming are important because they are two subsistence styles that represent the ancestors of billions of people around the world. Yet there are other important subsistence styles that are worth incorporating into this theory, such as herding and fishing^19,21^. We know less about the effects of other subsistence styles.

### Thousands of generations compared to one generation on a rice farm

It may be surprising that there were rice-wheat differences at all because these two farms have only existed for the length of a single person’s lifetime (roughly 70 years). The finding that rice-wheat differences can appear that fast suggests that direct experience on a farm is enough to influence people’s social style and way of thinking. In the scope of history, a single generation is an incredibly short period of time compared to the thousands of years rice has developed along with cultural institutions in southern China.

There is an important difference between these two farms and the rest of China. These two farms provide laboratories that expose people to farm work and the social relationships of the rice and wheat farms, but not the larger social institutions of the rice regions of southern China. For example, rice areas of southern China have more family clans than northern China^22^. There is evidence that regions with more clans are more collectivistic^23^. Over many generations, rice villages developed different social institutions, which probably magnified the underlying differences. Yet the state rice farm is an island of rice farming in northern China.

Its short existence and isolation from the social institutions of the south probably explain why differences between the rice and wheat farms were smaller than differences between southern and northern China as a whole. For example, holistic thought differences between the two state farms were 28% the size of differences between northern and southern China in a previous study^1^. This suggests that rice culture can form in the length of a single human’s lifetime, but we should not overlook the effects that accumulate as cultures build reinforcing institutions such as family clans, local dialects, and regional governments.

### Quasi-random assignment makes genetic explanations less likely

The quasi-random assignment makes it unlikely that there are systematic genetic differences between the rice and wheat farms. This helps put context around prior evidence of cultural differences in genetics tied to social traits across East and West^14^ and across rice and wheat regions in China^15^. Of course, the question of culture versus genes is not either-or. Genes evolve with cultural subsistence practices, such as in the classic finding that raising dairy cows selected for genes for lactose tolerance^24^. However, the quasi-random assignment makes it less likely that genetics are an explanation for the rice-wheat differences between these two farms.

### A window into East-West differences?

Psychologists have documented East-West cultural differences across decades of research^25^. Yet there is no consensus on why these cultures differ. Some researchers have theorized that East-West differences are rooted in differences in subsistence style^25^. The idea is that intensive farming—particularly rice farming—requires more interdependence than the wheat farming and herding of the West. Yet this explanation is hard to test because there are many differences between East and West in religion, language, government, and history. That makes it impossible to isolate just one causal factor.

The data from these two farms offer a potential insight into this larger East-West theory while practically eliminating these potential confounds across East and West. All three social and cognitive differences between these farms map onto differences previously documented between East and West^10,17,18^. The data from this study offers the clearest evidence yet that rice farming is a cause of collectivism—and perhaps a cause of East-West differences.

## Methods

### Farm samples

We aimed to test 100 farmers from each farm. Our stopping rule was to stop at 200, while still completing any sessions that were in the queue at the time of 200. The stopping rule was independent of any results. We waited until data collection was complete before analyzing the data. This resulted in a total sample of 234 farmers (Table 2 shows demographics).

We recruited farmers by working with division leaders in September and October, 2017. Each farmer belongs to a division on the farm and received information about the study from their division leader. Farmers then contacted our team to schedule a test session.

These tests were different from our study one year later of social comparison and happiness on the farms^9^. The test sessions and dependent variables for the two projects were separate. Our social comparison study did not test for any markers of collectivism. The sample sizes and demographics are different between the two studies.

A majority of the farmers in our sample (73.5%) grew up on the farms. Thus, most participants were lifelong farmers and descendants of people assigned to the farms. That leaves about a quarter of farmers (26.5%) who were assigned to the farms after childhood. Nearly all of the participants (96.2%) were farming in the year of the study. The rest were working on administrative tasks on the farms.

Farmers completed the tasks on paper individually. A research assistant was there to help with any questions. In the analyses, we controlled for potential demographic differences in age, gender identity, family income, and religion. We also controlled for maternal educational attainment because there is some evidence that it is a strong predictor of children’s outcomes^26^. We did not find any statistically significant cultural differences based on farmers’ own educational attainment (Supplementary Table S6). One potential explanation is that there was very little variance in educational attainment on the farms (Table 2).

### Measuring cultural differences

To measure cultural differences, we chose tests based on two criteria:

- First, we chose measures that previous research has linked to rice-wheat differences and larger East-West differences. We did this because it allows us to know whether the cultural differences between the rice and wheat farms are similar to larger East-West cultural differences established through decades of research^25^.
- Second, we chose implicit and non-self-report measures. This helps avoid the documented problems of using self-report surveys to measure cultural differences^27^. Meta-analyses have found that self-report scales fail to find East-West differences in collectivism^27,28^. One meta-analysis of self-report studies found people in Japan are more collectivistic than people in the US^28^. There is evidence that self-report collectivism is correlated with non-self-report measures in the wrong direction, even within a single country^4^. In contrast, studies using non-self-report and behavioral measures reliably find East-West differences^1,10,17,21,25^.

#### Implicit individualism

Farmers completed the sociogram task, which measures implicit individualism^17^. The task is implicit because we infer individualism through behavior, rather than asking participants to rate their individualism. Participants drew circles to represent the self and their friends in a diagram of their social network. Later, we measured the size of the self circle and the average of the friend circles (Fig. 2).

Prior studies have found that people in the US and Western Europe draw the self much larger than they draw friends, whereas self-inflation is lower in Japan and China^1,17^. Two studies have also found that people in wheat areas of China self-inflate more than people in rice areas^1,7^.

When testing the first few farmers, we discovered that they sometimes struggled to think of friends to draw on the sociogram task. We soon realized that the friend category is not as relevant for farmers in China as for college students who took this task in previous studies. After this discovery, we added a second sociogram task that asked farmers to draw family members. The farmers seemed to have no problems bringing to mind family members.

#### Loyalty/nepotism

Next, the farmers completed the loyalty/nepotism task^18^. The task asks people to imagine going into a business deal with a friend, who then lies during the deal, which causes the participant to make less money in the deal. Participants can punish the friend for their dishonesty by paying a small amount of money to delete money from their bank account (paying 0–100 RMB to delete 0–1000 RMB [US$148]). In another scenario, the friend is honest, and they can reward the friend by paying to add money to their bank account.

Crucially, participants completed two identical scenarios with a stranger. Thus, participants completed four scenarios in total (reward/punish friend/stranger). We analyzed whether participants treated the friend better than the stranger, even though the friend and the stranger acted the same. Treating the friend better could be seen positively as loyalty or negatively as nepotism.

We calculated whether participants treated the friend differently from how they treated the stranger as ([reward friend – punish friend] – [reward stranger – punish stranger]). Using this task, a previous study found that people in Singapore are more loyal/nepotistic than people in the US^18^. A study using real money found similar differences between the US and China in punishing in-groups, particularly when participants had time to think about their decisions^29^. Within China, two studies have found that people in rice areas are more loyal/nepotistic than people in wheat areas^1,7^.

The loyalty/nepotism task illustrates an important misunderstanding about collectivism. It is easy to bring to mind an intuitive picture of collectivism where people are generally friendly, nice, and pro-social. Yet we argue that collectivism is not about loving everyone^30^. Instead, collectivism is about tight social ties, filling social roles, and responsibilities to in close, trusted relationships. In contrast, ties are weaker for people outside of meaningful relationships, such as strangers.

#### Cultural thought style

We measured cultural thought style because previous studies have found more holistic thought in collectivistic cultures and more analytic thought in individualistic cultures^10,25^. Analytic thought focuses on individual components, formal logic, and abstract categories. In contrast, holistic thought focuses on the relationships between items, the context, and tolerance of contradiction. Although China tends to be holistic overall, holistic thought is more common in rice-farming areas of China than wheat-farming areas of China^1,7^.

We measured holistic thought with the 14-item picture version of the triad task^10^. In each triad, farmers saw a target object, such as the rabbit in Fig. 2. Then they chose one of two other objects to pair with it, such as a dog or carrot. One pairing belongs to the same abstract category (rabbits and dogs are mammals), and one pairing shares a functional relationship (rabbits eat carrots). We calculated the percentage of relational pairings as a measure of holistic thought. Previous studies have found that people in collectivistic cultures like China, Thailand, and India choose more relational pairings than individualistic cultures like the US, UK, and Netherlands^1,10,23^.

#### Wheat vs. rice

For the sake of simplicity, we use the term “wheat” to stand in for major dryland staples. We do this because the differences between rice and wheat are similar to the difference between rice and crops like millet, barley, corn, and potatoes^2,31^. Like the rest of northern China, the wheat farm also plants a portion of corn and other crops. The rice farm also farms portions of vegetables and other crops.

#### Test sessions

Completing the tasks and demographic questions took 30 to 40 min. Participants received a 30 Yuan mobile phone recharge card or a family-size jug of dishwashing detergent. Local farmers recommended these gifts as appropriate. Farmers took the tests at home or in an office. In the Supplementary Note 10, we find that the test setting was not significantly related to results for any of the cultural tests.

#### Analysis

We calculated descriptive statistics and Cronbach’s alpha using SPSS. We ran regression analyses and matching using the program R. We checked that the data met assumptions of statistical tests, including normality and equality of variance (Supplementary Methods Section 2). All *p* values are two tailed. This study was not pre-registered.

#### Propensity score matching

We used propensity score matching to reduce possible confounds from demographic differences in the sampling between the two farms. This helps address the shortcoming that our sampling in these hard-to-reach areas was not a random probability sampling. In the MatchIt package in R, we used optimal matching to create matched samples on age, gender identity, income, and maternal educational attainment.

All analyses use matching, except for Supplementary Table S3, which presents regressions without control variables or matching. Without control variables or matching, rice-wheat differences were non-significant for thought style (*B* = 0.07, *t*[227] = 0.64, *P* = 0.520, 95% CI [−0.14, 0.28], *r* = 0.04). If we include control variables (but without matching), the rice-wheat difference in thought style is significant (*B* = 0.28, *t*[214] = 2.28 *P* = 0.023, 95% CI [0.04, 0.53], *r* = 0.14). Thus, for thought style, it seems important to take into account the control variables (particularly age and maternal educational attainment).

Propensity score matching has advantages over just controlling for variables in regressions. For one, regressions assume that control variables have linear effects, whereas matching does not^32^. In addition, if there is a trait present in one group but not the other, propensity matching will exclude participants with that trait. This makes propensity score matching more conservative than controlling for demographics alone.

### Reporting summary

Further information on research design is available in the Nature Portfolio Reporting Summary linked to this article.

### Supplementary information

Supplementary Information

Reporting Summary

## Data Availability

The questionnaires and data are provided with this paper in the Open Science Framework 10.17605/OSF.IO/5TWCB and upon request from the first author.

## Code availability

Analysis scripts are provided with this paper in the Open Science Framework 10.17605/OSF.IO/5TWCB and upon request from the first author.
