# Supplementary material for: People quasi-randomly assigned to farm rice are more collectivistic than people assigned to farm wheat
Source: Nat Commun. 2024 Feb 27;15:1782. doi: 10.1038/s41467-024-44770-w (PMC10899190; doi:10.1038/s41467-024-44770-w)
Supplement: Supplementary file 1 — Supplementary Information [file 41467_2024_44770_MOESM1_ESM.pdf]

Supplementary Information

Table of Contents

Supplementary Methods

1. Language

2. Testing Assumptions of Statistical Tests

Supplementary Notes

1. Hui Participants

2. Chinese State Farms Relational Mobility Compared to 39 Studied Societies

3. Statistical Power

4. Holistic Thought Among Farmers in China Compared to Tea Farmers in Turkey

5. Livestock

6. Farm Samples and Backgrounds

7. No Evidence That Rice-Wheat Differences Are Weakening Over Time

8. Demographic Differences Between Rice and Wheat Farm Samples Before and After Propensity Score Matching

9. Farm Populations Over Time

10. No Statistically Significant Differences Based on Test Setting

11. Crops on the Farms

12. Farm Policies

13. Are the Small Environmental Differences Between Farms Meaningful?

14. Relational Mobility

Supplementary Figures and Tables

Figure S1: Relational Mobility on the Rice and Wheat Farms

Figure S2: Relational Mobility in the Farm Samples Compared to 39 Societies

Figure S3: Leftover Corn Straw on the Qukou State Farm

Table S1: Demographic Differences Between Rice and Wheat Farm Samples Before and After Propensity Score Matching

Table S2: Rice-Wheat Differences Remain After Controlling for Test Setting

Table S3: Rice-Wheat Differences Without Control Variables or Matching (Zero Order)

Table S4: Descriptive Statistics

Table S5: Descriptive Statistics for Binary Variables

Table S6: Farmers' Own Educational Attainment Is Not Significantly Related to Cultural Tasks

Supplementary Methods

1. Language

The research assistant communicated with the farmers in Mandarin Chinese. The farmers commonly understand Mandarin because the local dialect is close to Mandarin.

2. Testing Assumptions of Statistical Tests

We tested the assumptions of statistical tests by checking for skewness, kurtosis, and equality of variance using the raw data. For the four cultural difference measures, we calculated skewness and kurtosis then compared these to cutoffs of < 2 for skewness and < 7 for kurtosis.

Relational Pairings: Skewness = -2.01, Kurtosis = 3.84
Self-Inflation Friends: Skewness = 0.75, Kurtosis = 1.97
Self-Inflation Family: Skewness = 0.74, Kurtosis = 2.23
Loyalty/Nepotism: Skewness = 0.72, Kurtosis = 2.46

All four outcomes were below the cutoffs, except that the skewness of relational pairings was 0.01 over the recommended cutoff. Because the deviation from the cutoff was so small, we decided not to transform the data.

Next, we tested for equality of variances in each outcome across rice and wheat farms using Leven's test for equality of variances in the program R. The test reports whether there is significant evidence for inequality of variances. The result of each test was non-significant.

Supplementary Notes

1. Hui Participants

Ningxia is home to many Hui, who are a Muslim minority in China. According to the 2000 Census, Ningxia is 34% percent ethnic Hui, although only 6.5% of our sample was Hui. Compared to other Muslim populations like the Uyghurs, the Hui are quite assimilated into the majority Han culture, apart from religion. For example, most Hui speak Chinese[33].

We control for self-reported Hui identity in the analyses. The regressions in Table 3 found that Hui identity was not significantly related to self-inflation with family (*B* = -1.62, *t*[189] = -1.17, *P* = 0.242, 95% CI [-4.35, 1.10]), self-inflation with friends (*B*

= 1.09, $t[183] = 0.77$, $P = 0.445$, 95% CI [-1.69, 3.86]), loyalty/nepotism ($B = 0.16$, $t[189] = 0.12$, $P = 0.903$, 95% CI [-2.43, 2.75]), or holistic thought ($B = -0.30$, $t[181] = -1.29$, $P = 0.199$, 95% CI [-0.74, 0.17]).

2. Chinese State Farms Relational Mobility Compared to 39 Studied Societies

Although the relational mobility scale showed low reliability, we ran tentative comparisons to other published studies of relational mobility. Because of the low reliability, these comparisons should be interpreted with caution. Figure S1 shows the basic differences in relational mobility between the rice and wheat farms.

Figure S1

Relational Mobility on the Rice and Wheat Farms

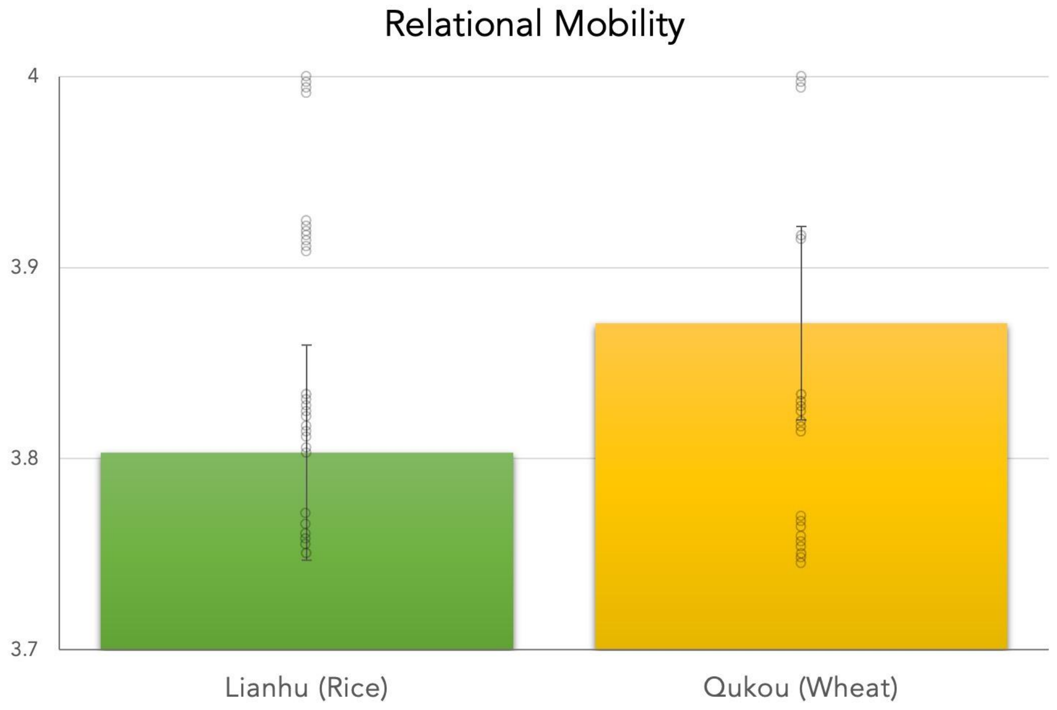

Note: Relational mobility on the rice farm (left) and wheat farm (right). Bars = 1 SEM. Dots = individual participants. Dots are displaced to avoid overlapping. The sample size was 192. This figure and Figure 3 use matched samples from propensity score matching to minimize demographic differences between the samples.

Next, we compared our relational mobility results to a recent study of 39 societies around the world[21]. We did this because we know of no prior study that has tested relational mobility in farming communities. Researchers have argued that farming is a low relational mobility subsistence style[21]. However, previous studies have relied on college samples or samples from Facebook[21]. Thus, our farmer data offers a rare opportunity to compare relational mobility in a farming community with the student and online samples common in psychology.

The farmers in China reported lower relational mobility (M = 3.84, SD = 0.57) than every other society tested (Figure S2). Note that this is a descriptive statistic. It is not a formal statistical test comparing this new data to the previous study in 39 cultures[21]. We suggest that future studies can check the robustness of this finding with other samples.

If farmers have lower relational mobility than all previous cultures studied, it would suggest two interesting implications for theory:

1. First, it would be consistent with the theory that farming is a low relational mobility subsistence style[21].
2. Second, it would be consistent with the idea that online samples common in psychology are giving us a narrowed view of the range of data across societies. This would suggest that widening our samples to include more diverse populations, such as this farming sample, would widen our view of human psychology.

This is echoed by two recent papers advocating for sample diversity in psychology. For example, one researcher argued recently that we should consider sample diversity beyond the WEIRD vs. non-WEIRD dichotomy[34]. For example, China is a non-WEIRD culture. But the difference in holistic thought between this farm sample and our previous university samples in China is nearly as large as the difference between our university samples in the US and China. This speaks to the benefit of getting samples not just outside the West, but with hard-to-reach populations.

Figure S2

Relational Mobility in the Farm Samples Compared to 39 Societies

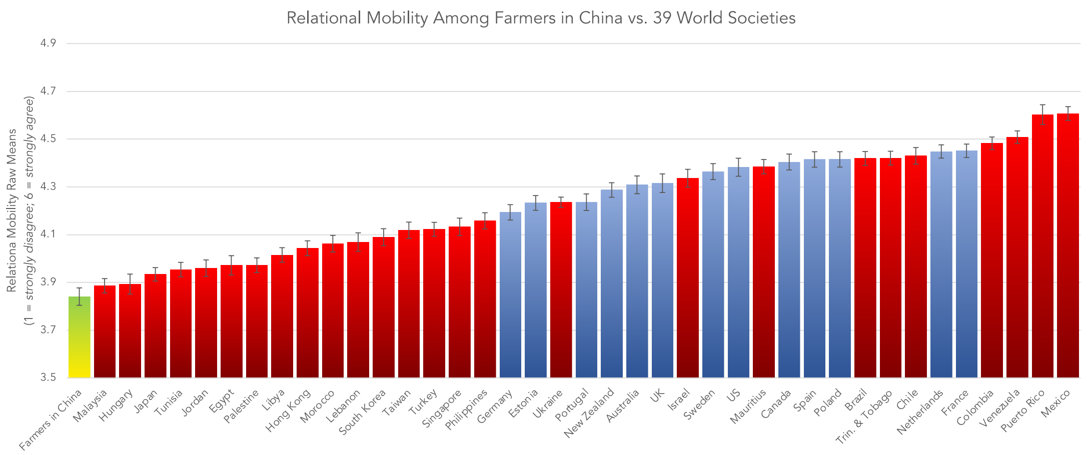

Note: This graph displays the average relational mobility among farmers in China in this study (far left bar) and in 39 societies tested in a global study[21]. Blue bars = Western cultures. Red bars = non-Western cultures, including former Soviet Bloc countries. Error bars = 1 SEM. Culture classifications come from Huntington (https://web.archive.org/web/20230108140249/https:/commons.wikimedia.org/wiki/File:Western_world_Samuel_P_Huntington.svg).

3. Statistical Power

We recruited as many farmers as we could approach given the limitations of our field site and recruitment. To give readers a sense of statistical power, we conducted a sensitivity analysis. With the final sample size of 234 participants, 80% statistical power, and two-tailed p values, the sample would be able to reliably detect effects as small as $r = 0.18$.

4. Holistic Thought Among Farmers in China Compared Tea Farmers in Turkey

Our holistic thought data can be compared to tea farmers in Turkey from a study that used the same task[18]. The Turkish study is the only other study we know of that tested farmers on this task. The farmers in Turkey from the previous study chose 68% holistic pairings[18]. Averaged across the rice and wheat farms, our participants averaged 87% holistic pairings. Note that this is a descriptive comparison. We cannot run a statistical comparison because the original study did not report the original data or enough statistical information for us to run a statistical test of these two samples.

Comparing different farming populations may be useful for subsistence theory.

The intensiveness of farming may be an important difference between farming systems. both rice and wheat farming in China are more intensive than wheat farming in Europe[2] and tea farming in Turkey. Thus, if this theory is correct, the more intensive forms of farming in China should produce more holistic thought than less intensive forms of farming. Of course, there are other differences between the farming communities in Turkey and China besides the intensity of the crops. Thus, we would need more research to test whether intensiveness is important.

Figure S3

Leftover Corn Straw on the Qukou State Farm

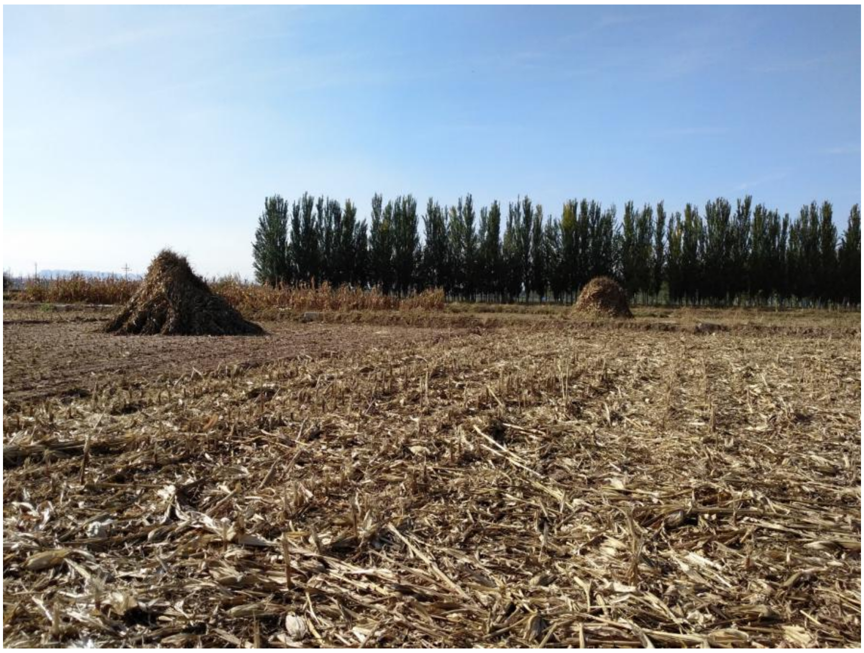

Note: The Qukou State Farm grows mainly dryland crops like wheat and corn (pictured), which require less labor than paddy rice and do not depend on irrigation. Photo by Xiawei Dong, November, 2017.

5. Livestock

Studies have found that herding cultures tend to be more individualistic than farming cultures[18,35]. In the two state farms, animal husbandry makes up only a small portion of the agricultural economy (12% in Lianhu and 8% in Qukou according to statistics we could find from 1988). Furthermore, this is livestock raising, rather than mobile herding. Livestock raising may be closer to farming than the mobile herding of cultures like the Mongolians and Tibetans.

6. Farm Samples and Backgrounds

We recruited farmers by working through division leaders on the farms in summer and fall of 2017. Each farmer belongs to a division, which has a division leader who oversees administrative tasks. Our team explained the study to the division leaders, and the leaders passed along the information to the farmers in their division. Farmers who were interested in participating contacted our team, and we set up a testing session.

Nearly all of the participants (96.2%) were currently farming (which we defined

as having farmed land in the year of the study). The other nine participants were helping with administrative tasks for the farm.

Most participants (73.5%) grew up on the farms (Lianhu or Qukou). That means that most of the participants were the descendants of farmers assigned by the government to the farms. That also means that most participants were lifelong farmers. However, about one-quarter (26.5%) were assigned to the farms after growing up elsewhere.

In this sample, the percentage of local born was slightly higher on the wheat farm (76.9%) than the rice farm (69.2%). This could make it seem like there are differences between the farms in the percentage of newcomers. However, we later returned to the farms to collect data for a separate project, and that sample showed the reverse pattern—more local-born people on the rice farm (79.8%) than the wheat farm (73.1%). Adding the two samples together reveals a nearly identical percentage of local-born farmers on the rice farm (74.5%) and wheat farm (75.2%). This is consistent with the data on population size of the two farms showing that the populations were mostly stable after 1980 (Supplementary Notes Section 9).

We ran regressions testing whether rice-wheat differences were larger or smaller for participants who were born on the farms. There are logical reasons to expect that differences could be in either direction. On the one hand, people born on the farms might be more enculturated because they are second generation. On the other hand, there is some evidence that cultural differences grow larger as people age[36], even into adulthood[37]. Thus, the original people assigned to the farms may have had more time to enculturate.

Regardless, among the tasks that showed significant rice-wheat differences in Table 3, we found no significant interactions that would support the idea that rice-wheat differences depended on whether people were born on the farms (tested as an interaction between rice farm and a dummy variable for born locally). Some of the means in the data were in opposite directions. For example, there was a non-significant interaction for self-inflation on the family sociogram in the direction that rice-wheat differences were larger among people not born on the farm ($B = 3.04$, $t[187] = 1.94$, $P = 0.054$, 95% CI [-0.05, 6.13]). However, loyalty-nepotism differences showed a non-significant trend in the opposite direction—slightly larger among people born on the farm ($B = 1.05$, $t[187] = 0.70$, $P = 0.486$, 95% CI [-1.92,

4.02]).

In sum, we found no significant interactions indicating that rice-wheat differences were larger or smaller for people born on the farm. The next section takes a different angle to this question: are rice-wheat differences larger or smaller among younger farmers? Analyzing age led to a similar conclusion.

One small note about the sample location: Ningxia is technically an autonomous region. However, for practical purposes, people in China think of it the same as other provinces. The same is true of other autonomous regions, like Xinjiang, Tibet, and Inner Mongolia.

7. No Evidence That Rice-Wheat Differences Are Weakening Over Time

The main text reports that the relational mobility questionnaire did not reach acceptable reliability. We suspected this could be because the questions are somewhat complex and difficult to understand, especially in an environment where people are not used to taking questionnaires. However, as living standards have risen in China, the younger farmers may have more exposure to writing, which could make the questionnaire easier for them to complete.

In line with this idea, we found that the scale reliability improved from 0.45 in the entire sample to 0.55 among farmers below 47 years old (the average age of the sample). In addition, rice-wheat differences were larger among younger participants than older participants (age x rice interaction in a regression: $B = 0.04$, $t[184] = 2.69$, $P = 0.008$, $r = 0.20$, 95% CI [0.01, 0.06]). This fits with the idea that comprehension was a problem for farmers with the relational mobility questionnaire.

We did not find significant interactions of age with rice for implicit individualism, holistic thought, or loyalty/nepotism. It is interesting that these results do not fit with the idea that rice-wheat differences are disappearing over time as China modernizes and the market economy reaches into more parts of Chinese society. Instead, rice-wheat differences were just as large among younger farmers.

8. Demographic Differences Between Rice and Wheat Farm Samples Before and After Propensity Score Matching

We used the MatchIt package in the program R (version 4.5.1) to do propensity score matching with optimal matching. This improves on the more basic method of nearest neighbor matching. Simple nearest neighbor matching finds the optimal match for the first participant, regardless of whether that leaves less-optimal matches for

subsequent participants. In contrast, optimal matching finds matches while trying to make the entire sample more optimally matched.

We ran *t* tests to compare demographic differences before and after matching the samples from the rice and wheat farms. We tested for differences in participants' self-reported gender identity, age, family income, and maternal educational attainment. Table S1 reports the results.

Table S1

Demographic Differences Between Rice and Wheat Farm Samples Before and After Propensity Score Matching

	Before Matching				After Matching			
	t	*P*	95% CI		*t*	*P*	95% CI	
Female	-1.92	0.056	-0.26	0.003	-0.44	0.662	-0.18	0.11
Age	1.72	0.086	-0.19	2.88	0.49	0.625	-1.23	2.03
Family Income	0.92	0.357	-0.13	0.37	0.49	0.622	-0.19	0.32
Maternal Education	0.69	0.489	-0.11	0.22	0.59	0.553	0.40	0.35
Degrees of Freedom:	219				186			

Note: This table tests for demographic differences between the samples from the rice farm and wheat farm before using propensity score matching (left) and after (right). Negative *t* values mean the rice farm scored lower than the wheat farm; positive values mean the rice farm scored higher. These tests are for the matching with the participants who completed the triad task. For the other tasks, the numbers differ slightly because of missing data. Analyses are independent samples *t* tests with two-tailed *p* values, without correction for multiple comparisons.

9. Farm Populations Over Time

In this section, we detail the history of the founding of the farms and their populations over time in more detail. The Lianhu rice state farm was formally established on August 16, 1955[10] ("到 8 月 16 日农场成立时", p. 34). The Qukou state farm was formally established a few months later in 1956[11] ("渠口高级农业生产合作社", p. 5).

This time period corresponds to the main growth period of China's state farms[38]. The government of the People's Republic of China started establishing state farms after the end of the civil war in 1949. The government established many farms in the 1950s, eventually peaking at 2,123 farms in 1962. The number of farms has stayed stable since then[38] (Table 1 from Zhang's paper).

We were able to locate yearly population records for the Lianhu rice farm through 2008. The farm population grew from its founding until reaching 5,960 people by the end of the Cultural Revolution in 1977. The statistics show that the

population of the farm was mostly stable from 1977 on, hovering around 6,000 people. The last data point shows 5,946 people in 2008. Although this data does not rule out the possibility of people moving in and out, it suggests that the farm population has been largely stable for the last 40 years.

The Qukou wheat farm population was similarly mostly stable around 7,000 people. The records from Qukou report population for every five years. Like Lianhu, Qukou grew from its founding through the end of the Cultural Revolution. Qukou had 6,975 people in 1980 and 6,607 people in 2000. Both farms experienced slight decreases in population after China instituted the One Child Policy in 1980.

In 2002, Qukou added a parcel of land called Taiyang Liang 15 kilometers from the main farm. The government used this land to settle farmers whose land in the Guyuan area of Ningxia suffered from desertification and ecological degradation. This increased the official population of the farm, but this area is separate from the main farm. When recruiting participants, we did not work with any team leaders in the Taiyang Liang area.

10. No Statistically Significant Differences Based on Test Setting

We tested most farmers (93.2%) in office rooms on the state farms. At the beginning of the study, we tested farmers at home, but we soon learned that the home environment was distracting. For example, we could not prevent other family members from entering the home during the tests. Therefore, we started testing the farmers in office rooms on the farms, where we could get a more quiet environment. A research assistant was present regardless of setting.

We analyzed the data to test whether the setting influenced the results. Table S2 reports regression analyses of whether the setting of the test (home or in an office) was related to the results. We used a dummy variable where 1 = the participant completed the tests at home and 0 = at an office.

The results show that test setting was not statistically significantly related to any of the outcomes. Differences in holistic thought style between people tested at home or in the office were not statistically significant (B = 0.32, t[180] = 1.27, P = 0.203, 95% CI [-0.15, 0.85]). Rice-wheat differences remained significant after controlling for test setting.

Table S2

Rice-Wheat Differences Remain Significant After Controlling for Test Setting

	B	*SE*	*t*	*P*	95% CI	
**Self-Inflation (Family Sociogram)**						
Female	-0.47	0.72	-0.65	0.520	-1.89	0.96
Age	-0.07	0.07	-1.14	0.256	-0.20	0.05
Hui	-1.60	1.39	-1.15	0.252	-4.33	1.14
Family Income	-0.25	0.37	-0.66	0.508	-0.98	0.49
Mother Education	-0.54	0.60	-0.90	0.370	-1.72	0.64
Tested at Home	-0.38	1.36	-0.28	0.779	-3.06	2.30
Rice Farm	-1.92	0.73	-2.62	0.010	-3.37	-0.47
**Self-Inflation (Friend Sociogram)**						
Female	-0.11	0.73	-0.15	0.884	-1.53	1.32
Age	0.03	0.07	0.48	0.632	-0.10	0.16
Hui	1.01	1.42	0.71	0.480	-1.78	3.79
Family Income	0.01	0.39	0.03	0.975	-0.75	0.78
Mother Education	0.17	0.61	0.27	0.784	-1.03	1.36
Tested at Home	1.10	1.34	0.82	0.416	-1.54	3.73
Rice Farm	-0.64	0.73	-0.87	0.385	-2.08	0.80
**Loyalty/Nepotism**						
Female	1.22	0.69	1.78	0.077	-0.13	2.57
Age	-0.04	0.06	-0.70	0.482	-0.17	0.08
Hui	0.13	1.32	0.10	0.922	-2.47	2.73
Family Income	-0.56	0.35	-1.59	0.114	-1.26	0.14
Mother Education	-1.25	0.57	-2.20	0.029	-2.38	-0.13
Tested at Home	0.43	1.34	0.32	0.747	-2.21	3.07
Rice Farm	1.83	0.70	2.63	0.009	0.46	3.21
**Holistic Thought**						
Female	0.03	0.12	0.24	0.809	-0.21	0.27
Age	0.07	0.01	6.36	< 0.001	0.05	0.09
Hui	-0.32	0.23	-1.38	0.167	-0.77	0.15
Family Income	0.06	0.07	0.83	0.408	-0.08	0.20
Mother Education	0.28	0.11	2.56	0.010	0.07	0.50
Tested at Home	0.32	0.25	1.27	0.203	-0.15	0.85
Rice Farm	0.29	0.13	2.27	0.023	0.04	0.54

Note: This table tests whether rice-wheat differences remained after controlling for whether participants took the tests in an office (0) or at home (1). Samples are matched using propensity score matching. Female, Hui, and Rice Farm are coded 0 = no, 1 = yes. Age is in years. Hui are a Muslim religious group in China. Degrees of freedom = 188, except 189 for the friend sociogram and 187 for holistic thought. Analyses are regressions with two-tailed *p* values.

11. Crops on the Farms and Rice Rotation

For shorthand, we call the Qukou farm the “wheat farm” and Lianhu the “rice farm.” As with northern and southern China as a whole, the cropland in the farms is not 100% rice or wheat. Lianhu also grows a portion of corn, wheat, silage (crops grown as feed for animals), and other crops. Qukou also farms other crops like corn, silage, and a small portion of rice on land near the Yellow River. We plan to write a follow-up paper exploring this small portion of land in more detail. In this paper, this piece of land is represented in the individual rice farming percentage analysis in Table 4.

In the year 2000, the state rice farm started to face shortages of irrigation water from the Yellow River. This forced the farm to start managing the insufficient amount of irrigation water. To do this, the farm started a rotation system. The farm assigns about one-third of farmers each year to farm dryland crops such as wheat. Then those farmers get to farm rice the next year, while another third of farmers get rotated into farming dryland crops. This process goes on year by year.

Importantly, the farm decides this in a top-down system designed to treat farmers equally. That means farmers do not self-select into farming rice or not. Instead, the farm rotates farmers away from rice for one year out of every three.

This creates an experiment within an experiment. The larger experiment is comparing the rice farm and the wheat farm. Within that experiment is the rotation of direct experience farming rice in the year we tested farmers. That allows us to test whether the direct experience farming in the year of testing is a stronger predictor of cultural differences than the overall rice and wheat farms (Table 4).

In line with the rotation system, roughly one-third of farmers in our sample on the rice farm (38.4%) did not farm rice in the year we tested them. The farmers who were not farming rice were farming dryland crops, such as wheat, corn, and sorghum. (These dryland crops are grown similarly.) Three participants were working primarily administrative roles on the farms, such as helping with accounting tasks (one participant on the wheat farm and two participants on the rice farm).

12. Farm Policies

In the main text, we discuss policies on the two farms. After the founding of these farms, arguably the biggest change to China’s farm policies was the move from collective profits to allowing family’s to profit from their own output (包产到户).

Because these two farms belonged to the same land reclamation group (农垦集团), they rolled out this policy within a year of each other. The first farmland to revert to family profit (土地承包) was in 1993 on Qukou and 1994 on Lianhu[10,11].

13. Are the Small Environmental Differences Between Farms Meaningful?

Although the rice and the wheat farm are similar on environmental variables (Table 1), some readers may wonder whether the remaining differences between the farms are large enough to be meaningful. In this section, we give readers a sense for the size of these differences by comparing the differences between the farms to differences across China as a whole.

Temperature: The difference in the average July temperature between the farms was 23.0 versus 23.6 C. For comparison, prefectures across China range from 8 to 33 C. That means a difference of 0.6 C is 2% of the range.

Precipitation: The farms differ 10 mm in precipitation. For comparison, prefectures across China range from 16 to 2,443 mm. That means a difference of 10 mm is less than 1% of the range (0.4%).

Elevation: Among 351 prefectures across China, elevation ranged from 3 to 4,748 meters. The difference between the land on most of the wheat farm versus the rice farm is about 50 meters. Fifty meters is 1% of the range for all of China.

Farm size: China's largest state farms are 67,000 hectares[38]. In contrast, China's average farm size is 0.65 hectares[39]. In comparison, the difference between these two farms (190 hectares) is under 1% of the range of farm sizes in China (0.3%).

14. Relational Mobility

In addition to the tasks in the main text, we also included the relational mobility questionnaire. This questionnaire asks people to rate the amount of freedom, flexibility, and choice people have in their social relationships in their society[21]. A study of 39 societies found that societies with a history of rice farming have lower relational mobility[21]. However, the farmers' responses were below the common cutoff of 0.60 for reliability ($\alpha = 0.45$).

We suspect farmers had difficulty with these questions, which are fairly abstract. For example, one question asks participants to imagine a conditional hypothetical: "If they did not like…they would…." On top of that, some items use negation (such as, "cannot freely choose"). Research has found that negatively worded items confuse survey respondents—even employees at the Census Bureau[40].

Because of the low reliability, we do not analyze the relational mobility questionnaire in the main text. However, the raw means showed that relational mobility was one standard error lower on the rice farm. In supplementary analyses, we found that younger farmers seemed to understand the questionnaire better (with a higher reliability), and rice-wheat differences were stronger among younger farmers (reported in Supplementary Notes Section 7). However, these results are open to question, given the low reliability of the scale.

Table S3

Rice-Wheat Differences Without Control Variables or Matching (Zero Order)

	B	*SE*	*t*	*P*	95% CI		*DF*
**Self-Inflation (Family Sociogram)**							
Rice Farm	-2.35	0.64	-3.68	< 0.001	-3.61	-1.09	224
**Self-Inflation (Friend Sociogram)**							
Rice Farm	-1.21	0.67	-1.80	0.073	-2.53	0.12	227
**Loyalty/Nepotism**							
Rice Farm	1.29	0.65	1.99	0.048	0.01	2.57	232
**Holistic Thought**							
Rice Farm	0.07	0.11	0.64	0.520	-0.14	0.28	227

Note: This table tests for rice-wheat differences without controlling for potential confounding variables or propensity score matching. Overall, results are similar to the main results. Rice-wheat differences remained significant for self-inflation on the friend and the family sociogram but became non-significant for holistic thought. Female, Hui, and Rice Farm are dummy variables where 0 = no, 1 = yes. Age is in years. Hui are a Muslim religious group in China sometimes considered an ethnicity. Farmers reported monthly family income per person from 1 (*1,000 Yuan and below*) to 11 (*10,000 Yuan and above*). Degrees of freedom differ slightly between tasks because of missing data. Analyses are regressions with two-tailed *p* values.

Table S4

Descriptive Statistics

	Minimum	Maximum	Mean	Std. Deviation
Proportion Relational Pairings	0.00	1.00	0.87	0.20
Self-Inflation Family	-12.67	25.60	3.04	4.93
Self-Inflation Friends	-12.00	22.00	4.12	5.07
Reward Friend	0.00	10.00	5.41	3.30
Punish Friend	0.00	10.00	3.22	3.45
Reward Stranger	0.00	10.00	4.50	3.32
Punish Stranger	0.00	10.00	3.04	3.31
Loyalty/Nepotism	-14.00	20.00	0.73	4.96
Relational Mobility	1.75	5.67	3.84	0.57
Age	28.00	63.00	46.99	5.66
Family Income per Capita	1.00	6.00	2.02	0.93
Mother Education	0.00	2.00	0.38	0.60
Individual Rice %	0.00	1.00	0.28	0.36

Note: Age is in years. Farmers reported their family monthly income per person in categories from 1 (*1,000 Yuan or less*) to 11 (*Over 10,000 Yuan*). Mother's educational attainment is coded as (0) no formal education, (1) elementary school, and (2) middle or high school. There were no responses for maternal educational attainment above high school.

Table S5

Descriptive Statistics for Binary Variables

	Minimum	Maximum	%
Female	0.00	1.00	61.4%
Hui	0.00	1.00	6.5%
Rice Farm	0.00	1.00	44.4%
Tested at Home	0.00	1.00	6.8%
Born on the Farm	0.00	1.00	73.5%

Note: These four variables are all coded such that 0 = no, 1 = yes. For example, Female is coded 0 = not female, 1 = female. Tested at Home represents whether the tests were in an office (0) or at home (1). Hui are a Muslim religious group in China, sometimes considered an ethnicity.

Table S6

Farmers' Own Educational Attainment Is Not Significantly Related to Cultural Tasks

	B	*SE*	*t*	*P*	95% CI		*DF*
**Self-Inflation (Family Sociogram)**							
Educational Attainment	-0.38	0.36	-1.06	0.292	-1.09	0.33	225
**Self-Inflation (Friend Sociogram)**							
Educational Attainment	0.58	0.37	1.58	0.115	-0.14	1.31	227
**Loyalty/Nepotism**							
Educational Attainment	0.18	0.36	0.51	0.614	-0.53	0.89	232
**Holistic Thought**							
Educational Attainment	0.08	0.06	1.34	0.180	-0.04	0.20	227

Note: This table uses generalized linear models to test for differences based on farmers' own educational attainment. Because the goal is not to compare the rice and wheat farms, this table does not use propensity score matching. The questionnaire asked participants to report their educational attainment from 1 (*elementary school*), 2 (*middle school*), 3 (*high school*), 4 (*vocational high school*), 5 (*vocational college*), 6 (*undergraduate degree*), 7 (*master's degree*), 8 (*PhD*). Sample sizes differ slightly between tasks because of missing data. Analyses are regressions with two-tailed *p* values.
