# Supplementary material for: People quasi-randomly assigned to farm rice are more collectivistic than people assigned to farm wheat
Source: Nat Commun. 2024 Feb 27;15:1782. doi: 10.1038/s41467-024-44770-w (PMC10899190; doi:10.1038/s41467-024-44770-w)
Supplement: Supplementary file 2 — Reporting Summary [file 41467_2024_44770_MOESM2_ESM.pdf]

nature portfolio

Corresponding author(s): Thomas Talhelm

Last updated by author(s): Sep 1, 2023

Reporting Summary

Nature Portfolio wishes to improve the reproducibility of the work that we publish. This form provides structure for consistency and transparency in reporting. For further information on Nature Portfolio policies, see our Editorial Policies and the Editorial Policy Checklist.

Statistics

For all statistical analyses, confirm that the following items are present in the figure legend, table legend, main text, or Methods section.

n/a	Confirmed	
☐	☒	The exact sample size (*n*) for each experimental group/condition, given as a discrete number and unit of measurement
☐	☒	A statement on whether measurements were taken from distinct samples or whether the same sample was measured repeatedly
☐	☒	The statistical test(s) used AND whether they are one- or two-sided<br>*Only common tests should be described solely by name; describe more complex techniques in the Methods section.*
☐	☒	A description of all covariates tested
☐	☒	A description of any assumptions or corrections, such as tests of normality and adjustment for multiple comparisons
☐	☒	A full description of the statistical parameters including central tendency (e.g. means) or other basic estimates (e.g. regression coefficient) AND variation (e.g. standard deviation) or associated estimates of uncertainty (e.g. confidence intervals)
☐	☒	For null hypothesis testing, the test statistic (e.g. *F*, *t*, *r*) with confidence intervals, effect sizes, degrees of freedom and *P* value noted<br>*Give P values as exact values whenever suitable.*
☒	☐	For Bayesian analysis, information on the choice of priors and Markov chain Monte Carlo settings
☐	☒	For hierarchical and complex designs, identification of the appropriate level for tests and full reporting of outcomes
☐	☒	Estimates of effect sizes (e.g. Cohen's *d*, Pearson's *r*), indicating how they were calculated

Our web collection on statistics for biologists contains articles on many of the points above.

Software and code

Policy information about availability of computer code

Data collection	No software was used
Data analysis	The programs SPSS versions 28, 29; R version 4.2.2, and the R package MatchIt 4.5.1 were used.

For manuscripts utilizing custom algorithms or software that are central to the research but not yet described in published literature, software must be made available to editors and reviewers. We strongly encourage code deposition in a community repository (e.g. GitHub). See the Nature Portfolio guidelines for submitting code & software for further information.

Data

Policy information about availability of data

All manuscripts must include a data availability statement. This statement should provide the following information, where applicable:

- Accession codes, unique identifiers, or web links for publicly available datasets
- A description of any restrictions on data availability
- For clinical datasets or third party data, please ensure that the statement adheres to our policy

The questionnaires and source data are provided with this paper in the Open Science Framework (DOI: OSF.IO/5TWCB) and upon request from the first author. Source data are provided with this paper.

Human research participants

Policy information about studies involving human research participants and Sex and Gender in Research.

Reporting on sex and gender	We report gender distribution of the samples and control for gender in the analyses.
Population characteristics	Table 2 reports participant demographics.
Recruitment	We aimed to test 100 farmers from each farm. We used convenience and snowball sampling because of the difficulty of recruiting participants on remote farms. Our stopping rule was to stop at 200, while still completing any sessions that were in the queue at the time of 200. The stopping rule was independent of any results. We waited until data collection was complete before analyzing the data. This results in a total sample of 234 farmers (Table 2 shows demographics).<br><br>We determined our sample size based on feasibility of recruitment. We recruited as many farmers as we could approach given the limitations of our field site and recruitment. The final sample size of 234 participants gives 90.6% statistical power to detect the average effect size in social psychology, r = 0.2136. This effect size is close to the effect size differences between the farms in this study (such as self-inflation r = 0.18).<br><br>Our rationale for choosing to study these samples of participants on these two farms is that the farms were set up with quasi-random assignment to the rice and wheat farms. This provides an opportunity to test for cultural differences based on rice versus wheat farming while minimizing confounds between rice and wheat farming. Our rationale for our method of sampling was that we tested adults because adults can provide informed consent to participate.<br><br>One limitation of this study is that there could be demographic differences such as age and education between the two farms. These samples are not representative of the population of China because they come from a specific region of China and because they come from farms. That means, for example, the participants do not come from urban areas. The samples are also not perfectly representative of the entire populations on the farms. For example, the farms include children below age 18, and we did not recruit children to participate.<br><br>Another related limitation is that we could not randomly sample farmers because of the difficulty of reaching participants in these remote farms. One limitation of using convenience sampling and snowball sampling is that recruitment is not random. Thus, participants who did not want to participate were not present in the sample. Participants who did not see announcements about the study also did not participate. This could pose challenges such as differences in the sample characteristics between the two farms. For example, one farm could have more female participants or older participants than the other farm. This could pose problems such as with maternal educational attainment, which was linked to lower holistic thought. If one farm sample has more participants with higher maternal educational attainment, it could artificially lower our estimates of holistic thought on that farm. To make up for this, we took three steps:<br><br>1. We measured a range of demographic variables for the farmers to measure variables that might otherwise be hidden confounds.<br>2. We statistically controlled for demographics in generalized linear models.<br>3. We used propensity score matching to create sub-samples from the rice and wheat farms that were more similar on potential demographic confounds.<br><br>These steps help minimize the possibility that demographic differences are causing spurious differences between the farms.
Ethics oversight	University of Chicago Institutional Review Board (SBS)

Note that full information on the approval of the study protocol must also be provided in the manuscript.

Field-specific reporting

Please select the one below that is the best fit for your research. If you are not sure, read the appropriate sections before making your selection.

☐ Life sciences ☒ Behavioural & social sciences ☐ Ecological, evolutionary & environmental sciences

For a reference copy of the document with all sections, see nature.com/documents/nr-reporting-summary-flat.pdf

Behavioural & social sciences study design

All studies must disclose on these points even when the disclosure is negative.

Study description	The data is quantitative. It is a natural experiment comparison of two groups--one rice farm and one wheat farm. We rely on the historical policy of pseudo-randomly assigning people to nearby rice and wheat farms in China to create a comparison between two groups.

Research sample	The sample is farmers on two state farms in the Chinese provinces of Ningxia. Table 2 reports the demographics. We aimed to recruit 100 farmers from each from. The samples are not purely random, although they present a natural experiment and are matched using propensity score matching.
Sampling strategy	We aimed to test 100 farmers from each farm. Our stopping rule was to stop at 200, while still completing any sessions that were in the queue at the time of 200. The stopping rule was independent of any results. We waited until data collection was complete before analyzing the data. This results in a total sample of 234 farmers.<br><br>One limitation of this study is that there could be demographic differences such as age and education between the two farms. Another related limitation is that we could not randomly sample farmers because of the difficulty of reaching participants in these remote farms. To make up for this, we took three steps:<br><br>1. We measured a range of demographic variables for the farmers to measure variables that might otherwise be hidden confounds.<br>2. We statistically controlled for demographics in generalized linear models.<br>3. We used propensity score matching to create sub-samples from the rice and wheat farms that were more similar on potential demographic confounds.<br><br>These steps help minimize the possibility that demographic differences are causing spurious differences between the farms.
Data collection	All of the tests and questionnaires are available on the Open Science Framework link. Participants took the questionnaires on pen and paper. A research assistant was present to help with understanding. Participants took the tests either at home or in an office. This variable is coded in the data, and it is analyzed in the supplemental materials. We found no meaningful effect of test setting.
Timing	Data was collected from the farms from July 3 to November 21, 2017.
Data exclusions	No participants were excluded from the analysis. However, propensity score matching will exclude participants in order to create matched samples. The exact sample sizes after matching are reported in the open source R output. Table S3 reports the results without propensity score matching.
Non-participation	No participants dropped out.
Randomization	This study is a natural experiment. The pseudo-randomization occured historically, when people were assigned to the farms. We rely on this "natural randomization" rather than randomization by us.

Reporting for specific materials, systems and methods

We require information from authors about some types of materials, experimental systems and methods used in many studies. Here, indicate whether each material, system or method listed is relevant to your study. If you are not sure if a list item applies to your research, read the appropriate section before selecting a response.

Materials & experimental systems

n/a	Involved in the study	
☒	☐	Antibodies
☒	☐	Eukaryotic cell lines
☒	☐	Palaeontology and archaeology
☒	☐	Animals and other organisms
☒	☐	Clinical data
☒	☐	Dual use research of concern

Methods

n/a	Involved in the study	
☒	☐	ChIP-seq
☒	☐	Flow cytometry
☒	☐	MRI-based neuroimaging
